# Supplementary material for: Microtubules soften due to cross-sectional flattening
Source: eLife. 2018 Jun 1;7:e34695. doi: 10.7554/eLife.34695 (PMC6053307; doi:10.7554/eLife.34695)
Supplement: Supplementary file 1. [file elife-34695-supp1.docx]

**Supplementary Table 1.** List of parameters used in the paper

Parameter Significance

*L* Effective length of filament (MT or flagellum)

*db* Optical bead separation

$\varepsilon$ Strain computed from optical bead separation

*s* Arc-length coordinate along the filament

$\hat{s}$ Normalized arc-length coordinate along the filament

*b* Optical bead radius

*F* Force required to deform the filament

*Fc* Classical buckling force for a beam

*B* Flexural rigidity

*R* Undeformed MT radius

*a* Semi-minor axis of MT cross-section

*e* Cross-sectional “eccentricty”

*N* Number of particles used to discretize a filament

$\gamma$ Friction coeffcient

$\tau_{relax}$ Mechanical relaxation time for filament

*Ec* Circumferential Young’s modulus

*Ea* Axial Young’s modulus

*G* Shear modulus

*MB* Critical bending moment at the onset of Brazier buckling

*h* MT “equivalent” thickness

*h*0 MT “effective” thickness

*p* (*lc* ) Rest (deformed) length of circumferential MT bonds in simulation

*d* (*la*) Rest (deformed) length of axial MT bonds in simulation

$\phi_{c}$ Local circumferential bending angle in simulation

$\phi_{a}$ Local axial bending angle in simulation

$\phi_{s}$ Local shear angle in simulation

*kc* Circumferential stretching spring constant

*ka* Axial stretching spring constant

$\kappa_{c}$ Circumferential bending “spring” constant

$\kappa_{a}$ Axial bending “spring” constant

$\kappa_{s}$ Shear “spring” constant
